# Clinical outcomes of parenchymal-sparing versus anatomic resection for colorectal liver metastases: a systematic review and meta-analysis

**DOI:** 10.1186/s12957-023-03127-1

**Published:** 2023-08-08

**Authors:** Kun Wang, Yin Liu, Mengdi Hao, Huimin Li, Xiaoqing Liang, Dajin Yuan, Lei Ding

**Affiliations:** Gastrointestinal Oncology Surgery, Beijing Shijitan Hospital, Capital Medical University, Beijing, 100038 China

**Keywords:** Parenchymal-sparing resection, Anatomic resection, Colorectal liver metastases, Outcomes, Meta-analysis

## Abstract

**Background:**

The advantages of parenchymal-sparing resection (PSR) over anatomic resection (AR) of colorectal liver metastases (CRLM) remain controversial. Here, we aim to evaluate their safety and efficacy.

**Methods:**

A systematic review and meta-analysis of short-term perioperative outcomes and long-term oncological outcomes for PSR and AR were performed by searching Pubmed, Embase, the Cochrane Library and Web of Science databases.

**Results:**

Twenty-two studies were considered eligible (totally 7228 patients: AR, *n* = 3154 (43.6%) vs. PSR, *n* = 4074 (56.4%)). Overall survival (OS, HR = 1.08, 95% CI: 0.95-1.22, *P* = 0.245) and disease-free survival (DFS, HR = 1.09, 95% CI: 0.94-1.28, *P* = 0.259) were comparable between the two groups. There were no significant differences in 3-year OS, 5-year OS, 3-year DFS, 5-year DFS, 3-year liver recurrence-free survival (liver-RFS) and 5-year liver-RFS. In terms of perioperative outcome, patients undergoing AR surgery were associated with prolonged operation time (WMD = 51.48 min, 95% CI: 29.03-73.93, *P* < 0.001), higher amount of blood loss (WMD = 189.92 ml, 95% CI: 21.39-358.45, *P* = 0.027), increased intraoperative blood transfusion rate (RR = 2.24, 95% CI: 1.54-3.26, *P* < 0.001), prolonged hospital stay (WMD = 1.00 day, 95% CI: 0.34-1.67, *P* = 0.003), postoperative complications (RR = 2.28, 95% CI: 1.88-2.77, *P* < 0.001), and 90-day mortality (RR = 3.08, 95% CI: 1.88-5.03, *P* < 0.001). While PSR surgery was associated with positive resection margins (RR = 0.77, 95% CI: 0.61-0.97, *P* = 0.024), intrahepatic recurrence (RR = 0.90, 95% CI: 0.82-0.98, *P* = 0.021) and repeat hepatectomy (RR = 0.64, 95% CI: 0.55-0.76, *P* < 0.001).

**Conclusion:**

Considering relatively acceptable heterogeneity, PSR had better perioperative outcomes without compromising oncological long-term outcomes. However, these findings must be carefully interpreted, requiring more supporting evidence.

**Trial registration:**

PROSPERO registration number: CRD42023445332.

**Supplementary Information:**

The online version contains supplementary material available at 10.1186/s12957-023-03127-1.

## Introduction

At present, colorectal cancer (CRC) ranks the third malignancy in both incidence and mortality worldwide [1]. Up to 50% of patients develop liver metastasis, and colorectal liver metastases (CRLM) have become the leading cause of mortality in patients with CRC [2]. Liver resection has been proved to be a promising cure opportunity for CRLM, with a 5-year survival rate of more than 50%. Nearly 20% of postoperative patients would survive for more than 10 years [3]. Which is an optimal surgical resection of CRLM, either anatomic resection (AR) or parenchymal-sparing resection (PSR), has been controversial. In general, the major goal for therapy is to achieve a negative surgical margin and to preserve as much liver parenchyma as possible [4]. AR can achieve radical resection of CRLM, especially for multifocal lesions or lesions invading large intrahepatic vessels. However, AR can cause more postoperative symptoms, including postoperative liver failure [5]. PSR excises the liver tumor with the minimally sufficient resection margin to preserve as much normal liver parenchyma and the major intrahepatic vessels as possible [6, 7]. PSR is equivalent to AR in oncological outcomes and correlates with lower postoperative morbidity and shorter hospital stay [8, 9]. However, major concerns have been raised about whether PSR could increase the positive rate of surgical margin and the risk of tumor recurrence [10, 11].

There has been no consensus on whether PSR is superior to AR for CRLM. Therefore, our purpose of this study was to compare the perioperative short-term and postoperative oncological long-term outcomes of CRLM treated with AR and PSR.

## Materials and methods

### Literature search strategy

This meta-analysis was performed according to the PRISMA guidelines [12]. All analyses were based on previously published studies and therefore did not require ethical approval or informed consent. In order to ensure accuracy and to minimize deviation, literature retrieval, literature screening, data extraction and quality evaluation were carried out by two scientific investigators independently. A systematic literature search was conducted on medical databases PubMed, Embase, the Cochrane Library, and Web of Science to select articles that compared CRLM patients undergoing AR with PSR surgery, until January 2022. Literature retrieval was not limited by the language, type or geographical area. Specific search strategies were developed for each database using the following keywords and/or MeSH terms: “anatomic*” OR “major” OR “extended”; “nonanatomic*” OR “parenchyma* sparing” OR “wedge” OR “minor” OR “limited”; (“Colorectal Neoplasms” AND “Neoplasm Metastasis”) OR “Colorectal liver metastases*” OR “CRLM/CLM”.

### Inclusion and exclusion criteria

**Fig. 1:**
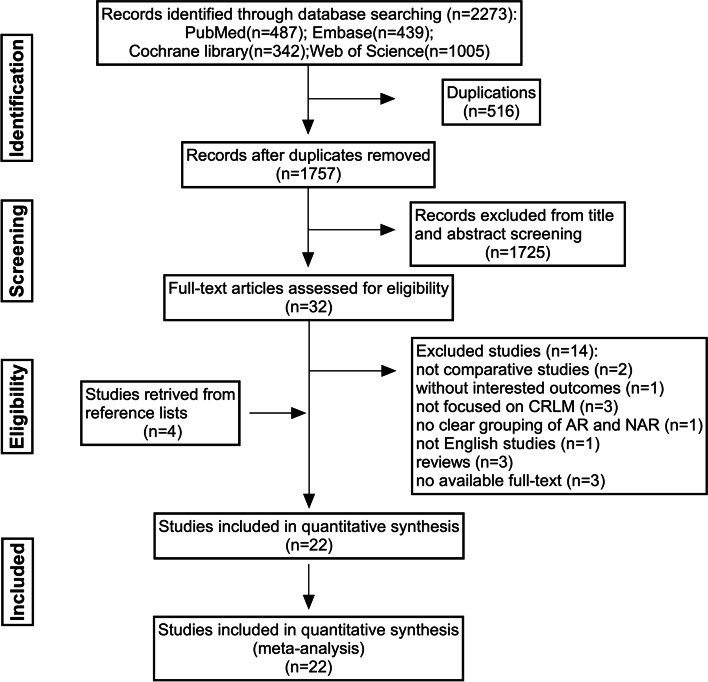
PRISMA flowchart of the study selection

In order to ensure the reliability, candidate studies were determined according to the following inclusion and exclusion criteria. Inclusion criteria included: (1) pathologically diagnosed with CRLM and treated with surgery; (2) comparing AR with PSR, where resection approach was considered as a variable in survival analysis; (3) perioperative short-term and long-term survival outcomes; (4) human studies; (5) sample size, follow-up time, literature language: unlimited. Studies met the above inclusion criteria were included in the meta-analysis. Exclusion criteria included: (1) unfocused CRLM, AR and PSR were not clearly grouped; (2) single-arm AR or PSR studies; (3) perioperative or survival outcomes were not reported or could not be extracted; (4) non-comparative studies such as reviews, letters, case reports, and meeting abstracts; (5) full text was not available. Studies that met one of the above exclusion criteria were excluded. The detailed literature search strategy was described in Fig. 1.

### Data extraction

The preliminary selected studies were classified and managed using Endnote X9 software. An Excel sheet was created to collect relevant data of all included studies. The data were extracted independently by two investigators. If there were disagreements, the teams were jointly resolved to reach an agreement. Long-term oncological outcomes were the primary endpoints of this meta-analysis, including overall survival (OS), disease-free survival (DFS), and liver recurrence-free survival (Liver-RFS). Secondary endpoints were perioperative outcomes, including duration of surgery, amount of blood loss, intraoperative blood transfusion rate, hospital stay, postoperative complications, positive resection margin, 90-day mortality, intrahepatic recurrence, and repeat hepatectomy. Study characteristics (publication year, first author, number of patients), patient characteristics (age, gender), and tumor characteristics (primary site, number of metastases, size of metastases, simultaneous resection, and CEA level) were also collected.

### Quality assessment

The quality of non-randomized controlled trials was evaluated by the modified Newcastle–Ottawa Scale (NOS) [13]. Two investigators independently assessed the quality of the literature. The NOS scale assesses three quality parameters (patient selection, intergroup comparability and outcome assessment) that were divided into eight specific items. There are slight differences in scoring case–control and cohort studies. The total score shall be 9 stars, the higher the score, the better the quality, while seven stars or more are considered as high quality. Low-quality studies with a NOS score less than five stars were excluded. The detailed quality evaluation of the included literature was listed in Table S1.

### Statistical analysis

The meta-analysis was performed by using Stata (version 14.0, Stata Corp) software. For continuous variables, weighted mean differences (WMD) and 95% confidence interval (CI) were used for evaluation. When the mean and standard deviation (SD) were not reported, calculations were made according to the equation proposed by Hozo et al. [14]. Survival data were used as dichotomous variables at different time points (3-year, 5-year OS, DFS, liver-RFS) with relative risk (RR) and 95% CI. When no specific survival data were reported, we estimated specific survival rate using the Kaplan-Meier plots. Meanwhile, we calculated the cumulative hazard ratio (HR) and 95% CI for each study from the Kaplan–Meier plots using the Engauge digitizer software (version 11.3) [15]. The overall effect was determined using the z-test. A 2-sided *p*-value < 0.05 was considered statistically significant.

Statistical heterogeneity between studies would be explored by examining forest plots and by using the X^2^ test. When the degree of CI overlap between studies in the forest plot was relatively low, we would consider that heterogeneity existed in the included studies. A p < 0.10 in the X^2^ test indicated the existence of heterogeneity. Heterogeneity was quantified using the I^2^ statistic. The I^2^ values of 25-49%, 50-74%, and 75-100%, could be interpreted as low, moderate, and high heterogeneity, respectively. If I^2^ < 50%, fixed effects model would be applied, otherwise, random effects model would be applied.

### Sensitivity analysis and publication bias

Sensitivity analysis was performed using Stata software (version 14.0, Stata Corp) to explore the robustness of the results and potential sources of heterogeneity. For each study being removed, a new meta-analysis was performed. If heterogeneity was significantly reduced, this specific study was considered to be a major source of heterogeneity and further evaluation was required. Publication bias was assessed by funnel plot and was quantified by Egger test. A p > 0.1 indicated that there was no publication bias [16].

## Result

### Included studies and quality assessment

The entire process of the literature search was described in Fig. 1. A total of 2273 articles were retrieved from the databases. After excluding duplicates and title-abstract, 32 articles were included in the full-text analysis. According to the selection criteria, 14 articles were further excluded, while another 4 articles were included by references. All literature quality assessment NOS scores were ≥ 7 (Table S1). Finally, 22 studies were included in this meta-analysis [8–10, 17–35]. A total of 7228 patients with CRLM who underwent liver resection from 1980 to 2019 were included, of which 4074 (56.4%) underwent PSR and 3154 (43.6%) underwent AR.

### Characteristics of the included studies

The baseline characteristics of the included studies were summarized in Table 1, including gender, age, primary tumor site, the number of metastases, the size of metastases, synchronous resection, and the CEA level. In AR and PSR, males accounted for 58.7% (1469/2503) and 60.7% (2207/3638), respectively. The proportion of males in the PSR group was slightly higher than that in the AR group. In most studies, the mean or median age was around 65. Among 4702 primary tumor sites, 3198 (68%) were in the colon whereas 1504 (32%) in the rectum. In the AR group, 1168 (69.8%) of the 1673 tumors were located in the colon, whereas 505 (30.2%) in the rectum. In the PSR group, 2030 (67%) of the 3029 tumors were located in the colon, whereas 999 (33%) in the rectum. The mean follow-up time for the studies ranged from 0 to 235 months.

**Table 1:** Characteristics of the included studies

First Author (Year)	Enrollment period	Country	Group	No. of patients	Gender (Male%)	Age	Primary tumor (Colon/ Rectum)	Number of metastases	Largest metastasis size(cm)	Synchronous liver metastases (%)	CEA level (ng/mL)	Type of survival outcomes	Follow-up (months)	NOS
Andreou 2021 [9]	2012–2019	Switzerland	AR	13	9(69.2%)	61(30–79)	9/4	N/A	N/A	9(100%)	N/A	OS, RFS	55	8
			PSR	79	51(64.6%)	62(36–84)	59/20	N/A	N/A	45(57%)	N/A			
Dam 2014 [25]	1991–2010	The Netherlands	AR	129	67(51.9%)	64(24–82)	79/50	4(1–12)	3.0(0.0–20.0)	N/A	N/A	OS, DFS	33(0–235)	8
			PSR	169	110(65.1%)	64(28–88)	98/71	1(1–3)	3.0(0.4–13.0)	N/A	N/A			
DeMatteo 2000 [17]	2001–2013	Italy	AR	148	88(59%)	< 70:110(74%); > 70:38(26%)	110/38	> 1:30(20%)	≥ 5 cm:48(32%)	N/A	< 200:101(92%); ≥ 200:9(8%)	OS	25(1–140)	7
			PSR	119	67(56%)	< 70:83(70%); > 70:36(30%)	96/33	> 1:23(19%)	≥ 5 cm:19(16%)	N/A	< 200:84(90%); ≥ 200:9(10%)			
Donadon 2018 [31]	2001–2013	Italy	AR	110	67(60%)	63.8 ± 10.2	N/A	3.5 ± 2.6	4.9 ± 2.5	44(40%)	N/A	OS, DFS	33(1–83)	8
			PSR	110	74(67%)	61.9 ± 11.0	N/A	3.7 ± 3.3	4.5 ± 2.5	43(39%)	N/A			
Finch 2007 [21]	1993–2003	UK	AR	280	171(61%)	63(26–84)	N/A	2(1–14)	4.5(0.7–20)	117(41.8%)	18 ng/dl(1–37,140)	OS, DFS	33(24–144)	7
			PSR	96	64(67%)	63(24–79)	N/A	1(1–9)	3.3(0.4–15)	36(38%)	5 ng/dl(1–12,124)			
Guzzetti 2008 [22]	1996–2005	Italy	AR	102	58(56.8%)	< 70:78(76.4%); > 70: 24(23.6%)	55/21	Single 59(61.4%); Multiple 37(38.6%)	> 5 cm:31(32.3)	N/A	< 200:52(89.6); ≥ 200:6(10.4)	OS, DFS	N/A	8
			PSR	106	63(59.5%)	< 70: 77(72.6%); > 70: 29(27.4%)	60/31	Single 67(64.4%); Multiple 37(35.6%)	> 5 cm:21(20.8)	N/A	< 200:51(86.4); ≥ 200:8(13.6)			
Hosokawa 2017 [29]	2000–2015	France	AR	242	141(58%)	64.0 ± 10.0	158/84	N/A	2.04 ± 0.8	121(50%)	159.7 ± 1,347.6	OS, RFS	41	8
			PSR	1478	888(60%)	64.1 ± 11.0	980/498	N/A	1.93 ± 0.76	721(49%)	65.7 ± 303.0			
Joechle 2020 [34]	2006–2016	USA	AR	105	69(65.7%)	54(29–82)	72/33	1(1–9)	2.0(0.16–5.3)	75(71%)	N/A	OS, RFS, liver-RFS	43.1	7
			PSR	105	71(67.6%)	56(26–79)	82/23	1(1–8)	1.8(0.1–5.8)	81(77%)	N/A			
Kokudo 2001 [18]	1980–1999	Japan	AR	96	54(56.3%)	58.7 ± 1.0	71/25	Single:54(56.3%); ≥ 3:20(20.8%)	5.81 ± 0.4	46(47.9%)	N/A	OS	N/A	7
			PSR	78	46(59%)	60.3 ± 1.2	49/29	Single:42(53.8%); ≥ 3:19(24.4%)	2.69 ± 0.16	56(71.8%)	N/A			
Lalmahomed 2011 [24]	2000–2008	Netherlands	AR	88	56(64%)	65(30–82)	55/33	2(1–7)	4(1–15)	35(40%)	> 200:10(12%)	OS, DFS	35(1–111)	8
			PSR	113	70(62%)	65(36–86)	59/54	1(1–7)	3(1–7)	43(38%)	> 200:6(5%)			
Lordan 2017 [30]	2000–2010	UK	AR	238	130(54.6%)	64.8(24–86)	N/A	Single:161(67.7%); Mutiple:76(31.9%)	3.2(0.4–20)	14 (5.9)	N/A	OS, DFS	36(0.12–144)	8
			PSR	238	135(56.7%)	65.7(31–87)	N/A	Single:153(64.3%); Mutiple:85(35.7%)	3.1(0.5–14)	15 (6.3)	N/A			
Matsuki 2016 [26]	2005–2013	Japan	AR	23	17(74%)	62(29–84)	15/8	5(1–17)	2.0(0.5–3)	13(57)	4.9(0.6–230)	OS, RFS, Liver-RFS	40(5–81)	8
			PSR	40	25(63%)	64(40–81)	26/14	4(1–27)	1.8(0.5–3)	18(45)	4.2(0.5–117)			
Matsumura 2016 [27]	1999–2012	Japan	AR	32	22(68.8)	62.5(27–80)	22/10	7(4–31)	3.0(0.8–5)	N/A	9.35(0.6–975)	OS, RFS, Liver-RFS	N/A	7
			PSR	113	74(65.5)	60(40–81)	65/48	6(4–33)	2.5(0.4–5)	N/A	7(0.5–3097)			
Memeo 2016 [28]	2006–2013	France	AR	266	145(55%)	61(29–82)	189/77	4(3–15)	3.3(0.6–20)	127(48%)	N/A	OS, DFS	N/A	8
			PSR	266	146(55%)	62.4(40–80)	194/72	4(3–9)	3.5(0.6–11)	134(50%)	N/A			
Mise 2016 [8]	1993–2013	Houston	AR	144	80(56%)	58(22–87)	105/39	N/A	1.9(0.3–3.0)	17(12%)	2.9(0.4–250.3)	OS, RFS, Liver-RFS	37(2—208)	8
			PSR	156	94(61%)	60(30–88)	113/43	N/A	1.5(0.4–3.0)	50(32%)	2.5(0.4–430.9)			
Okumura 2019 [10]	2004–2017	France	AR	82	51(62.2%)	64(43–85)	55/27	2(1–8)	2.8(0.5–13)	50(61%)	N/A	OS, RFS, Liver-RFS	33.9(6–120)	8
			PSR	82	50(61%)	65(33–83)	48/34	2(1–7)	2.5(0.5–15)	45(54.9%)	N/A			
Pandanaboyana 2018 [32]	1993–2011	UK	AR	582	N/A	< 65:282(48.5%); > 65:300(51.5%)	N/A	2	4.5(3–7)	294(50.5%)	N/A	OS, DFS	32.2(17.5–56.9)	8
			PSR	409	N/A	< 65:175(42.8%); > 65:234(57.2%)	N/A	2	2.7(2–4)	228(55.7%)	N/A			
Sarpel 2009 [23]	1987–2007	USA	AR	94	54(57%)	60.8 ± 10.4	60/10	1.7 ± 1.2	6.6 ± 4.7	8(8%)	N/A	OS, DFS	34	8
			PSR	89	51(57%)	62.3 ± 11.6	59/12	1.4 ± 1.0	3.5 ± 2.3	8(9%)	N/A			
She 2020 [35]	1990–2017	China	AR	70	38(54.3%)	61.0(29–85)	N/A	1(1–7)	2.45(1.0–11.0)	38(55.1%)	8.8(0.7–802)	OS, DFS	39.8(2.9–183.9)	8
			PSR	70	47(67.1%)	61.0(31–85)	N/A	1(1–multiple)	2.5(0.9–11.0)	27(38.6%)	8.85(1–526)			
Spelt 2018 [33]	2006–2014	Sweden	AR	60	39(65%)	65(61–69)	N/A	3(2–5)	2.65(1.7–4.0)	38(64.4%)	5(3–18)	OS	35	7
			PSR	59	35(59.3%)	69(63–76)	N/A	2(2–4)	2.2(1.5–3.0)	29(51.8%)	5(3–17)			
Stewart 2004 [19]	1988–2001	UK	AR	69	N/A	62(23–79)	N/A	1–3:65(97%); > 3:2/67(3%)	< 5 cm:14(23.3%); > 5 cm:46(76.7%)	N/A	625U/l(14–308,040)	OS	N/A	7
			PSR	27	N/A	64(28–82)	N/A	1–3:22(91.7%); > 3:2(8.3%)	< 5 cm:14(77.7%); > 5 cm:4(22.3%)	N/A	105U/l(1–2662)			
Zorzi 2006 [20]	1991–2004	Italy	AR	181	113(62.4%)	< 65:120(66.3%); > 65:61(33.7%)	113/46	Single:99(54.7%); Multiple:82(45.3%)	3(0.3–18)	73(40.3%)	< 200:160(88.4%); > 200:7(3.9%)	OS	25	7
			PSR	72	46(64%)	< 65:44(61%); > 65:28(39%)	42/17	Single:45(62.5%); Multiple:27(37.5%)	2.1(0.5–6)	25(34.7%)	< 200:63(87.5%); > 200:1(1.4%)			

*Abbreviations*: *AR* Anatomic resection, *DFS* Disease-free survival, *N/A* Not available, *OS* Overall survival, *PSR* Parenchymal-sparing resection, *RFS* Recurrence‐free survival

### Overall survival (OS)

**Fig. 2:**
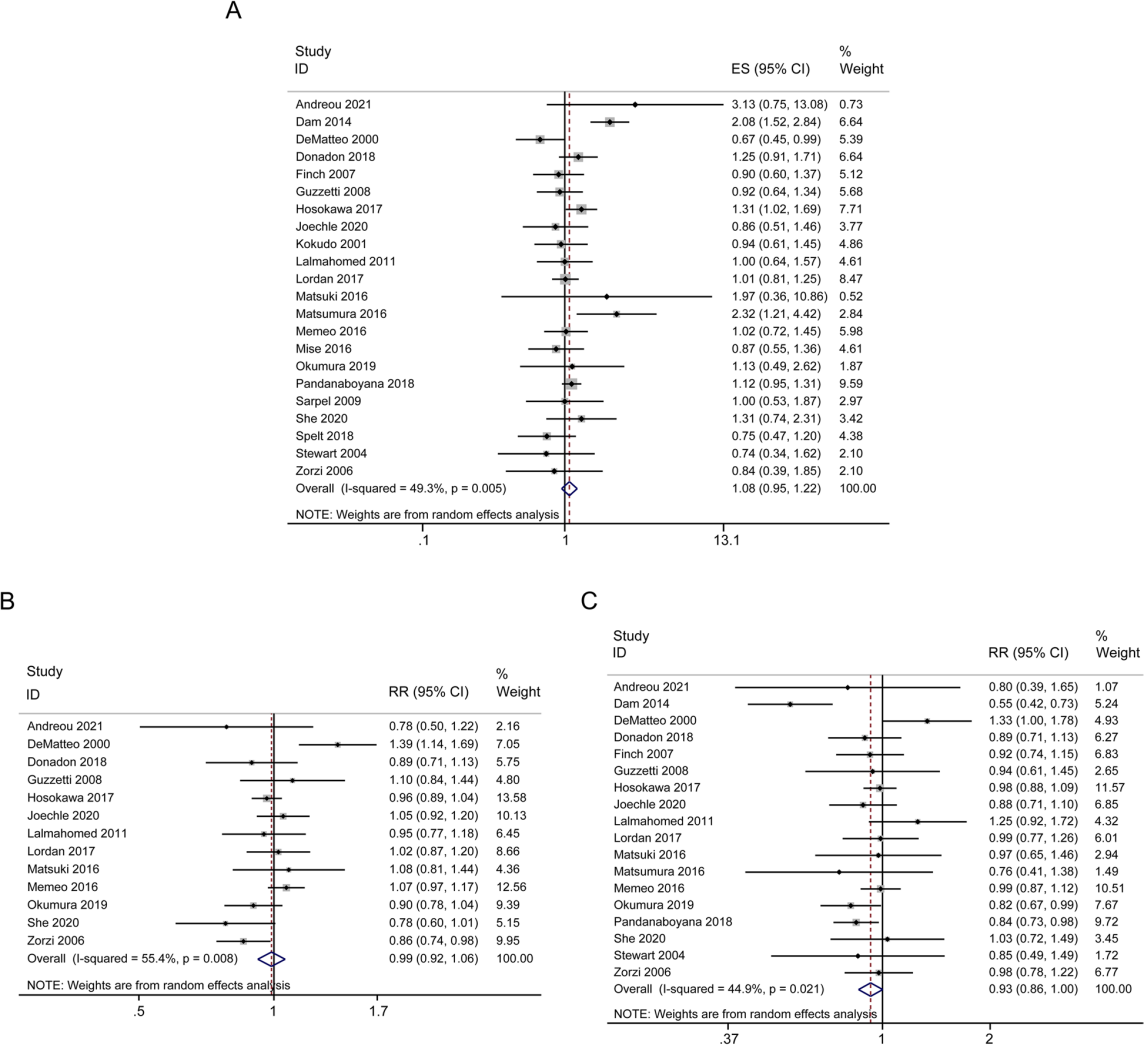
Forest plots of the effect of AR versus PSR on overall survival (OS). Cumulative hazard ratio (HR) of overall survival (OS) (**A**), risk ratio (RR) of 3-year OS (**B**), and 5-year OS (**C**). HR and RR are presented with 95% CI

The primary long-term outcome of OS was summarized in Table 2 and Fig. 2. HR-values extracted from 22 studies were incorporated into the assessment of OS. However, no clear evidence of any benefit of PSR on survival was found (HR = 1.08; 95% CI, 0.95-1.22; *p* = 0.245; I^2^ = 49.3%), as shown in Fig. 2A. The 3-year OS was comparable between AR and PSR groups (RR = 0.99; 95% CI, 0.92-1.06; *p* = 0.728; I^2^ = 55.4%) (Fig. 2B). The 5-year OS was slightly higher in PSR group than that in AR group (RR = 0.93; 95% CI, 0.86-1.00; *p* = 0.054; I^2^ = 44.9%) (Fig. 2C). The studies were moderately heterogeneous and used a random effect model.

**Table 2:** Results of meta-analysis comparing AR and PSR for CRLM

		Patients				Study heterogeneity		
Outcomes of interest	Studies	AR	PSR	WMD/RR/HR (95% CI)	P value	I^2^(%)	P value	Effect model
**Long-term**								
Overall survial (OS)	22	3154	4074	1.08(0.95–1.22)	0.245	49.3	0.005	Random
3‐year OS	13	1668	2878	0.99(0.92–1.06)	0.728	55.4	0.008	Random
5‐year OS	18	2760	3692	0.93(0.86–1.00)	0.054	44.9	0.021	Random
Disease‐free survival (DFS)	14	2260	3368	1.09(0.94–1.28)	0.259	75.1	< 0.001	Random
3‐year DFS	10	1237	2581	0.98(0.89–1.07)	0.66	0	0.897	Fixed
5‐year DFS	14	2260	3368	0.88(0.73–1.07)	0.212	70.1	< 0.001	Random
3‐year Liver-RFS	5	386	496	1.02(0.9–1.15)	0.789	0	0.79	Fixed
5‐year Liver-RFS	5	386	496	1.00(0.88–1.14)	0.981	0	0.592	Fixed
**Short‐term**								
Duration of operation (min)	13	1234	1364	51.48(29.03–73.93)	< 0.001	98.6	< 0.001	Random
Estimated blood loss (mL)	10	886	992	189.92(21.39–358.45)	0.027	98.6	< 0.001	Random
Intraoperative blood transfusion	12	1799	3004	2.24(1.54–3.26)	< 0.001	74	< 0.001	Random
Length of hospital stay (day)	15	1813	3011	1.00(0.34–1.67)	0.003	66.6	< 0.001	Random
Positive margin (mm)	12	1244	1080	0.77(0.61–0.97)	0.024	33.9	0.119	Fixed
Postoperative complications	12	1880	3116	2.28(1.88–2.77)	< 0.001	0	0.639	Fixed
90‐day mortality	7	1711	2826	3.08(1.88–5.03)	< 0.001	0	0.796	Fixed
Intrahepatic recurrence	15	2299	2036	0.90(0.82–0.98)	0.021	26.2	0.166	Fixed
Repeat hepatectomy	14	2362	3398	0.64(0.55–0.76)	< 0.001	12.4	0.317	Fixed

*Abbreviations*: *AR* Anatomic resection, *CI* Confidence interval, *CRLM* Colorectal liver metastases, *DFS* Disease-free survival, *HR* Hazard ratio, *RR* Risk ratio, *OS* Overall survival, *PSR* Parenchymal-sparing resection, *RFS* Recurrence‐free survival, *WMD* Weighted mean difference

### Disease-free survival (DFS)

**Fig. 3:**
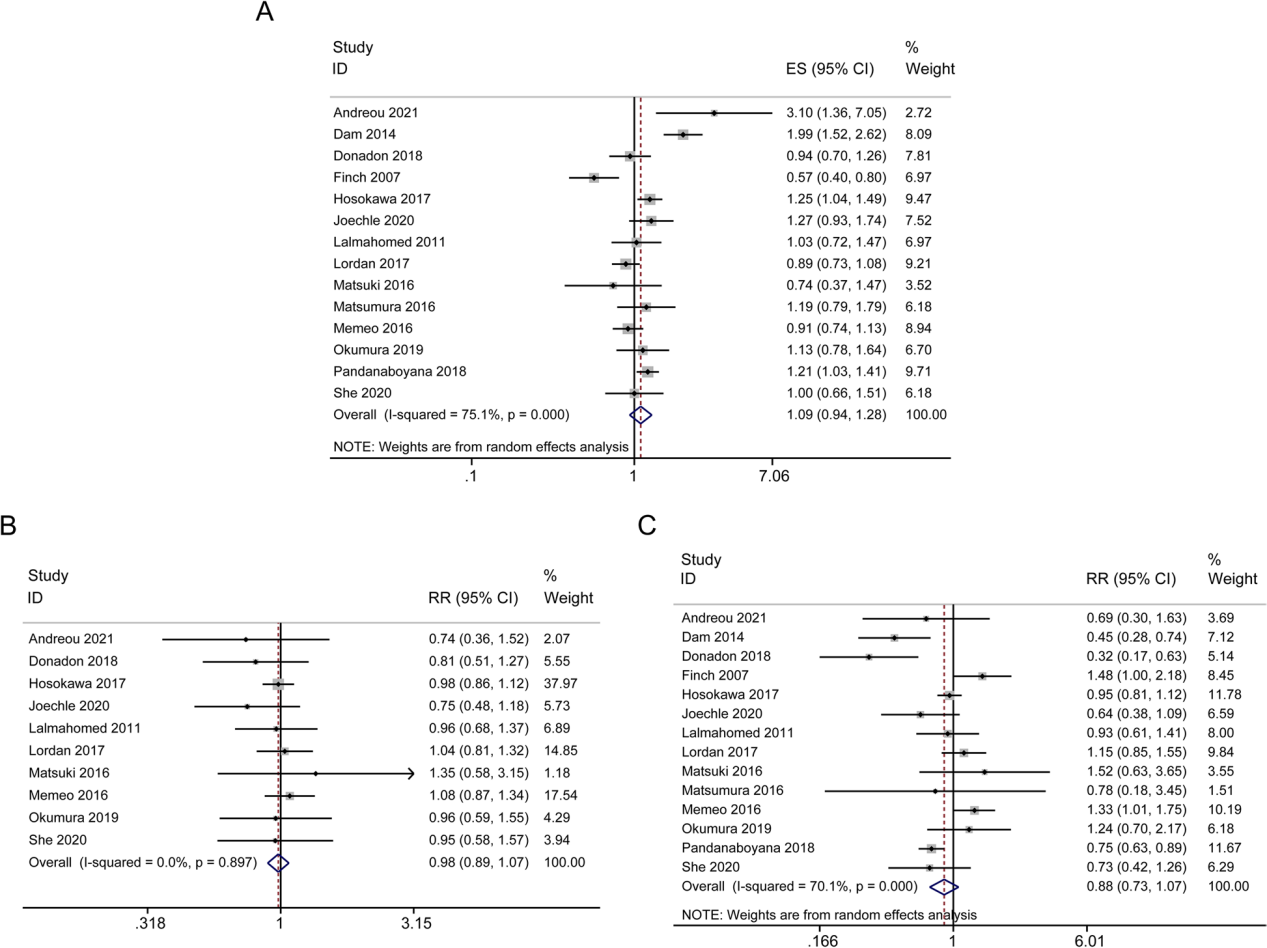
Forest plots of the effect of AR versus PSR on disease-free survival (DFS). Cumulative hazard ratio (HR) of DFS (**A**), risk ratio (RR) of 3-year DFS (**B**), and 5-year DFS (**C**)

The primary long-term outcome of DFS was summarized in Table 2 and Fig. 3. HR-values extracted from 14 studies were incorporated into the assessment of DFS, moderate heterogeneity was observed among these studies, the random effect model was applied. The combined effect was HR = 1.09; 95% CI, 0.94-1.28; *p* = 0.259. The related forest plots were shown in Fig. 3A. 3-year DFS was reported in 10 studies, low heterogeneity was observed among these studies (I^2^ = 0.0%, p = 0.897). The fixed effect model was applied, the combined effect was RR = 0.98; 95% CI, 0.89-1.07; *p* = 0.660 (Fig. 3B). The combined effect of 5-year DFS was RR = 0.88; 95% CI, 0.73-1.07; *p* = 0.212 (Fig. 3C). There was no significant difference in DFS between the AR group and the PSR group.

### Liver recurrence-free survival (Liver-RFS)

Results from 5 included literatures showed that 3-year liver-RFS (RR = 1.02; 95% CI, 0.90-1.15; *p* = 0.789; I^2^ = 0.0%) and 5-year liver-RFS (RR = 1.00; 95% CI, 0.88-1.14; *p* = 0.981; I^2^ = 0.0%) in both AR and PSR procedures were comparable, as shown in Fig. S1A-B.

### Short-term outcomes

**Fig. 4:**
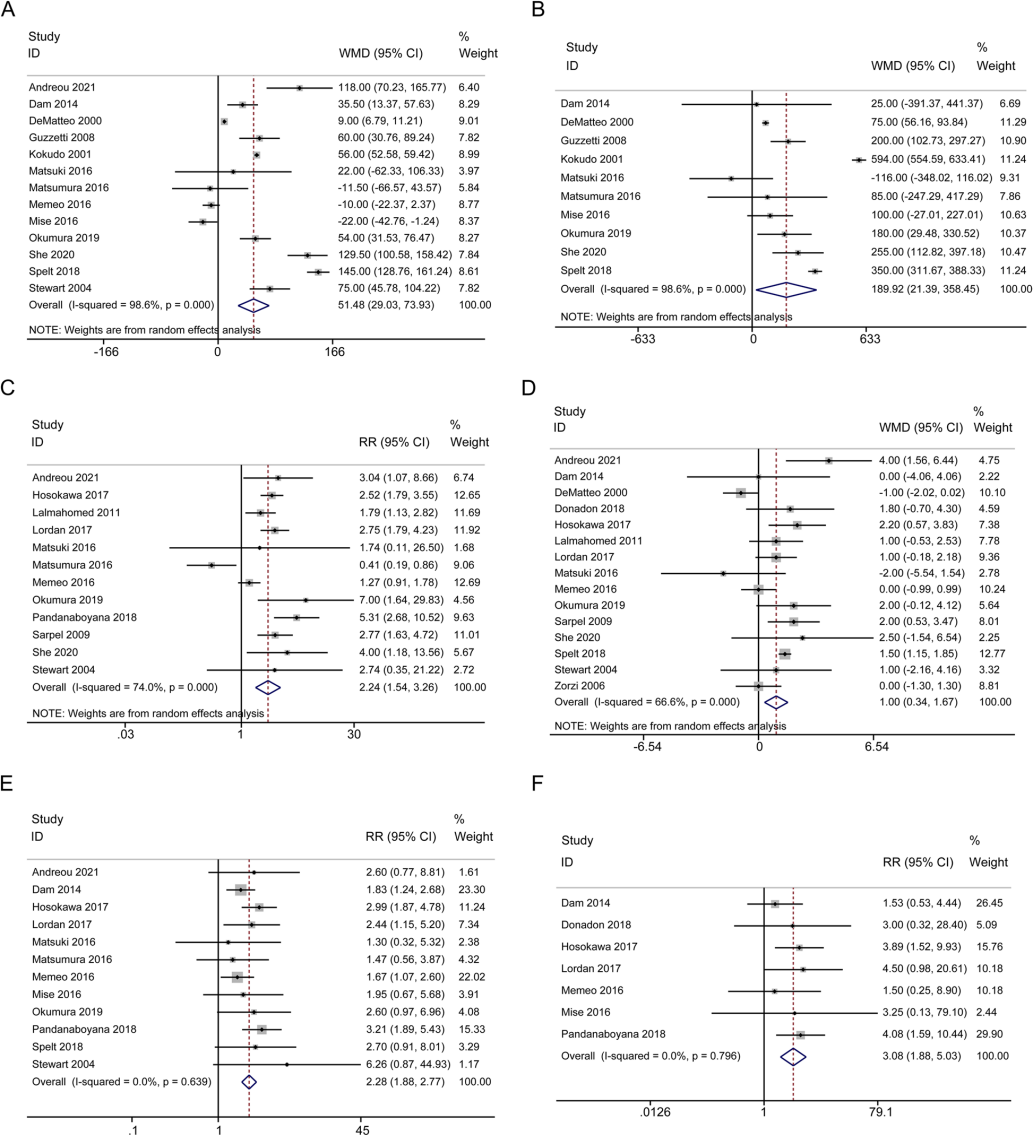
Forest plots of potential effects of AR versus PSR on short-term outcomes. Duration of operation (**A**), estimated blood loss (**B**), intraoperative blood transfusion (**C**), length of hospital stay (**D**), postoperative complications (**E**), and 90-day mortality (**F**)

**Fig. 5:**
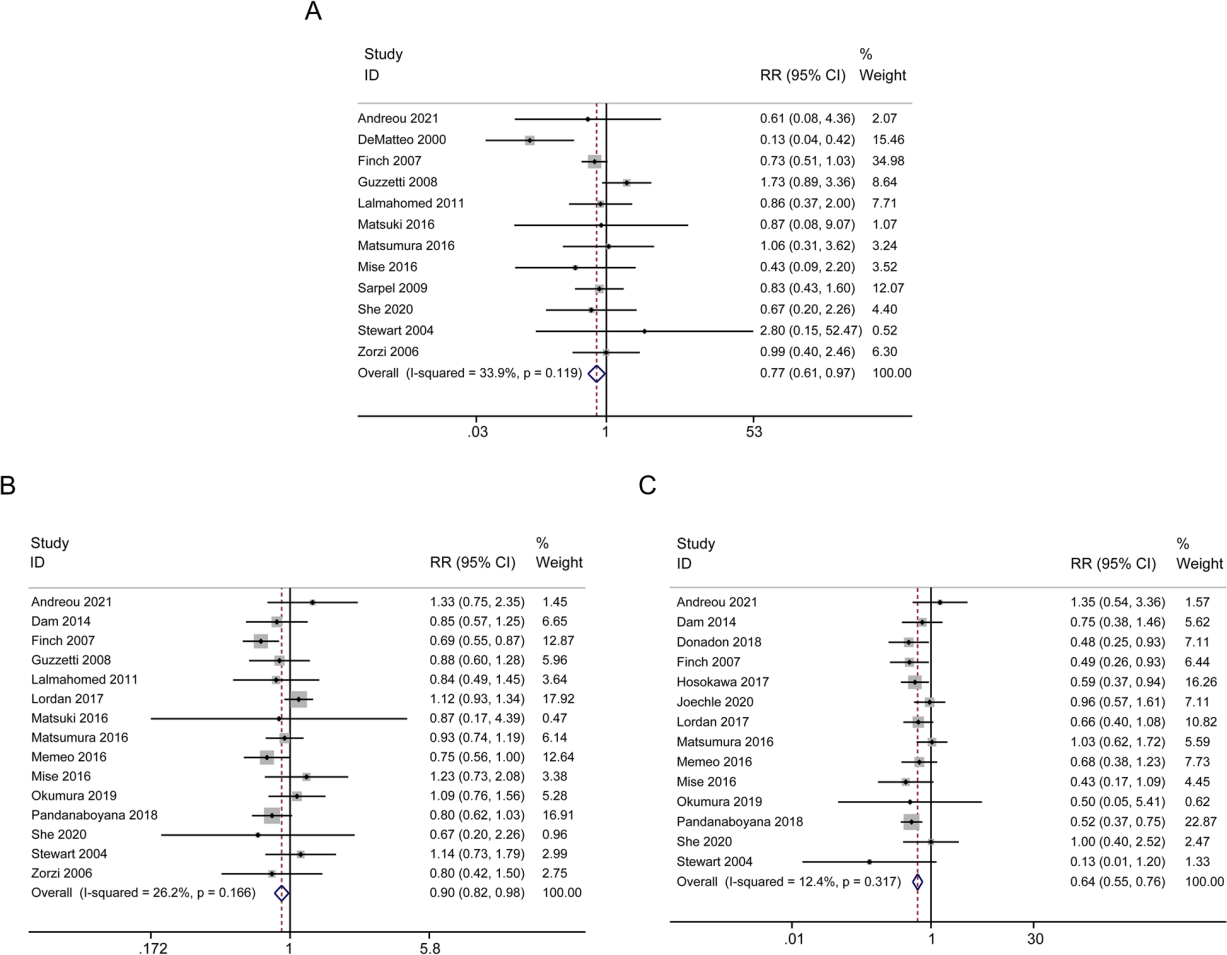
Forest plots of potential effects of AR versus PSR on short-term outcomes. Positive margin (**A**), intrahepatic recurrence (**B**), and repeat hepatectomy (**C**)

Short-term outcomes included duration of operation, blood loss, intraoperative blood transfusion rate, length of hospital stay, postoperative complications, 90-day mortality, positive resection margin, intrahepatic recurrence, and repeat hepatectomy. As summarized in Table 2, Fig. 4 and Fig. 5, compared with PSR group, AR group was associated with longer operative time (13 studies, WMD = 51.48 min; 95% CI, 29.03-73.93; *p* < 0.001; I^2^ = 98.6%, Fig. 4A), higher amount of blood loss (10 studies, WMD = 189.92 ml; 95% CI, 21.39-358.45; *p* = 0.027; I^2^ = 98.6%, Fig. 4B), increased intraoperative blood transfusion rate (12 studies, RR = 2.24; 95% CI, 1.54-3.26; *p* < 0.001; I^2^ = 74.0%, Fig. 4C), prolonged hospital stay (15 studies, WMD = 1.00d; 95% CI, 0.34-1.67; *p* = 0.003; I^2^ = 66.6%, Fig. 4D), increased postoperative complications (12 studies, RR = 2.28; 95% CI, 1.88-2.77; *p* < 0.001; I^2^ = 0.0%, Fig. 4E) and increased 90-day mortality (7 studies, RR = 3.08; 95% CI, 1.88-5.03; *p* < 0.001; I^2^ = 0.0%, Fig. 4F). 12 studies showed that PSR group was associated with a higher rate of positive resection margin (RR = 0.77; 95% CI, 0.61-0.97; *p* = 0.024; I^2^ = 33.9%, Fig. 5A). 15 studies indicated that intrahepatic recurrence was more obvious in PSR group (RR = 0.90; 95% CI, 0.82-0.98; *p* = 0.021; I^2^ = 26.2%, Fig. 5B). 14 studies suggested a higher repeat hepatectomy rate in PSR group (RR = 0.64; 95% CI, 0.55-0.76; *p* < 0.001; I^2^ = 12.4%, Fig. 5C). In terms of short-term outcomes, there were certain differences between the PSR and AR groups.

### Sensitivity analysis and publication bias

Sensitivity analysis of long-term and short-term outcomes obtained robust results. No significant changes in effect values were observed after sequentially removing one study compared to the overall analysis. Funnel plots were used to display publication bias. A symmetrical distribution of funnel plots could be observed. The publication bias test (Egger) confirmed that there was no publication bias in all included studies (Fig. S2-3).

## Discussion

Hepatectomy is a well-established treatment option for CRLM, in an attempt to achieve complete tumor resection while preserving sufficient residual healthy liver parenchyma to limit the risk of postoperative liver dysfunction/failure [36]. AR is recommended for therapeutic resection when liver metastases are relatively large or multiple, or when tumors invade the portal veins. However, extensive liver resection may be associated with post-hepatectomy liver failure [37]. PSR has been increasingly recognized as an appropriate and effective treatment in recent years, however, whether the non-anatomical nature of PSR leads to recurrence and worse long-term outcomes remains debated.

This meta-analysis has been focused on the differences in perioperative and long-term outcomes between AR and PSR for CRLM therapy. We retrospectively analyzed 7228 patients with CRLM from 22 independent studies. Our results indicated that compared with AR, PSR had better perioperative prognosis, shorter operative time, less intraoperative bleeding, a lower blood transfusion rate, fewer postoperative complications, and reduced 90-day mortality. Despite this benefit, there was a slightly higher incidence of positive margin, an increased risk of postoperative intrahepatic recurrence, and an increased rate of repeat resections for PSR. Long-term outcomes, including OS, DFS and liver-RFS, were comparable between AR and PSR without significant differences. We performed an updated meta-analysis and added new outcomes, such as positive resection margin, intrahepatic recurrence, and repeat hepatectomy rate, which is an innovation of this meta-analysis.

AR was likely to remove undetected micro-metastases and to obtain adequate tumor-free margins. The risk of postoperative recurrence of PSR was generally limited, while AR may not have a preventive effect on intrahepatic or extrahepatic recurrence [18, 31]. Recent studies have demonstrated that PSR is preferred for the treatment of resectable CRLM when permitted by the tumor size and location, without increasing the risk of remnant liver recurrence, associated with lower postoperative morbidity and shorter hospital stay, and with an equal oncological outcome [9, 38–40]. Burlaka et al. confirmed that parenchyma-sparing surgery should be a priority pathway for complex treatment of patients with deeply located lesions of the right liver lobe and bilobar liver metastases [41].

At present, the biggest question about PSR is whether it increases intrahepatic recurrence, which is an important predictor of survival outcome of CRLM patients after hepatectomy. Given that the goal of PSR is to minimize resection of the normal liver without sacrificing oncologic outcome, an increased risk of intrahepatic recurrence and a higher rate of repeat resections may not be surprising. In patients with small solitary CRLM, parenchymal-sparing hepatectomy (PSH) has no negative effect on OS, RFS, Liver-RFS, and does not increase the recurrence of liver remnants. However, in the case of liver recurrence, salvage repeat hepatectomy after PSR improves 5-year survival rate in patients with recurrence [8, 10, 42–44]. The greatest advantage of PSR lies in the increased treatment options after recurrence, especially the increased chance of reoperation, resulting in prolonged survival. Therefore, if PSR is performed first, with sufficient remnants of healthy liver tissues, repeat hepatectomy can be associated with improved OS [8, 43, 45]. For patients with more than 6 lesions, the survival time of patients with PSR was significantly longer than that of patients with major hepatectomy [46]. In a short-term and long-term study of 1720 patients with liver tumors < 30 mm in the right lobe who underwent PSH or right-lobe hepatectomy, 5-year RFS and OS were comparable between the two groups. However, repeated hepatectomy was performed more frequently in PSH. And the 5-year OS in PSH group was significantly higher than right-lobe hepatectomy group [29]. These data suggest that PSR has better oncologic benefit for repeat hepatectomy in the setting of recurrence. Our meta-analysis results showed that patients treated with PSR had certain risk of intrahepatic recurrence, however, the two procedures yielded comparable results in terms of 3-year and 5-year liver-RFS. The 5 year-OS was increased in PSR group by parenchyma-sparing repeat hepatectomy. Therefore, this meta-analysis helps to strengthen the application of PSR in CRLM. In the surgical decision-making of CRLM, it is necessary to ensure the radical resection of all lesions, retain as much liver parenchyma as possible, with little impact on liver function and low postoperative complications, and can increase the possibility of second resection after recurrence, thereby improving survival.

However, this meta-analysis has several limitations. Although we have conducted an extensive review of the literature available, all the included studies were non-randomized, single-center studies, which introduced selection bias and the risk of non-sufficient clinical evidence. Second, the choice of resection methods and patients’ baseline parameters may influence the analytic results. Our study collected 7228 CRLM patients from 22 studies, as an update of the current discussion of AR and PSR surgical outcomes.

In conclusion, our meta-analysis suggests that PSR has comparable safety and efficacy to AR, with favorable perioperative outcomes without compromising oncological outcomes. However, high-quality multicenter randomized controlled trials are needed in the future to validate the robustness of our findings.

### Supplementary Information

**Additional file 1:** **Fig. S1.** Forest plots of the effect of AR versus PSR on 3-year liver recurrence-free survival (liver-RFS) (A) and 5-year liver-RFS (B). **Fig. S2.** Funnel plots of cumulative OS (A), 3-year OS (B), 5-year OS (C), cumulative DFS (D), 3-year DFS (E), 5-year DFS (F), 3-year liver-RFS (G), and 5-year liver-RFS (H). **Fig. S3.** Funnel plots of short-term outcomes. Duration of operation (A), estimated blood loss (B), intraoperative blood transfusion (C), length of hospital stay (D), postoperative complications (E), 90‐day mortality (F), positive margin (G), intrahepatic recurrence (H), and repeat hepatectomy (I).

**Additional file 2:** **Table S1.** Quality of studies evaluated by modified Newcastle-Ottawa scale.

**Additional file 3.** **Supplemental file. **The full electronic search strategy for each database.

## Data Availability

The data analyzed during the current study are available from the corresponding author on reasonable request.
