# Supplementary material for: Clinical outcomes of parenchymal-sparing versus anatomic resection for colorectal liver metastases: a systematic review and meta-analysis
Source: World J Surg Oncol. 2023 Aug 8;21:241. doi: 10.1186/s12957-023-03127-1 (PMC10408219; doi:10.1186/s12957-023-03127-1)
Supplement: Supplementary file 1 — Additional file 1: Fig. S1. Forest plots of the effect of AR versus PSR on 3-year liver recurrence-free survival (liver-RFS) (A) and 5-year liver-RFS (B). Fig. S2. Funnel plots of cumulative OS (A), 3-year OS (B), 5-year OS (C), cumulative DFS (D), 3-year DFS (E), 5-year DFS (F), 3-year liver-RFS (G), and 5-year liver-RFS (H). Fig. S3. Funnel plots of short-term outcomes. Duration of operation (A), estimated blood loss (B), intraoperative blood transfusion (C), length of hospital stay (D), postoperative complications (E), 90‐day mortality (F), positive margin (G), intrahepatic recurrence (H), and repeat hepatectomy (I). [file 12957_2023_3127_MOESM1_ESM.docx]

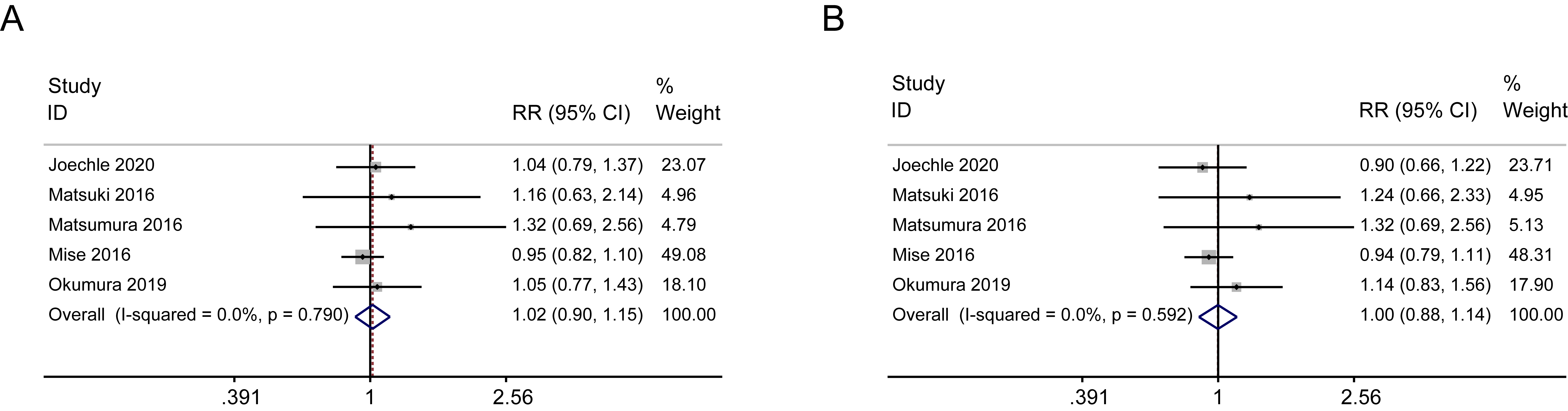
 **Fig.S1** Forest plots of the effect of AR versus PSR on 3-year liver recurrence-free survival (liver-RFS) (A) and 5-year liver-RFS (B).


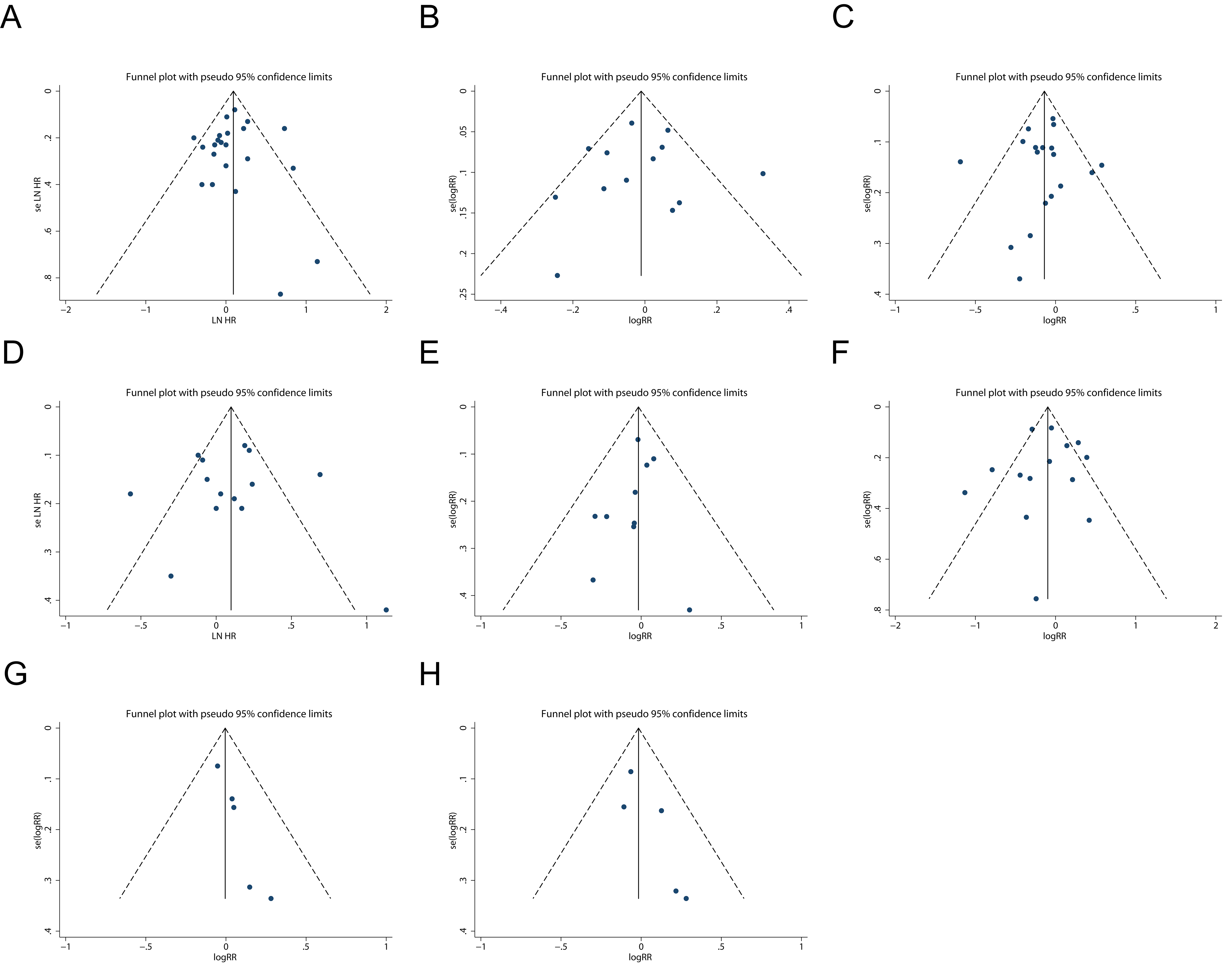


**Fig.S2** Funnel plots of cumulative OS (A), 3-year OS (B), 5-year OS (C), cumulative DFS (D), 3-year DFS (E), 5-year DFS (F), 3-year liver-RFS (G), and 5-year liver-RFS (H).


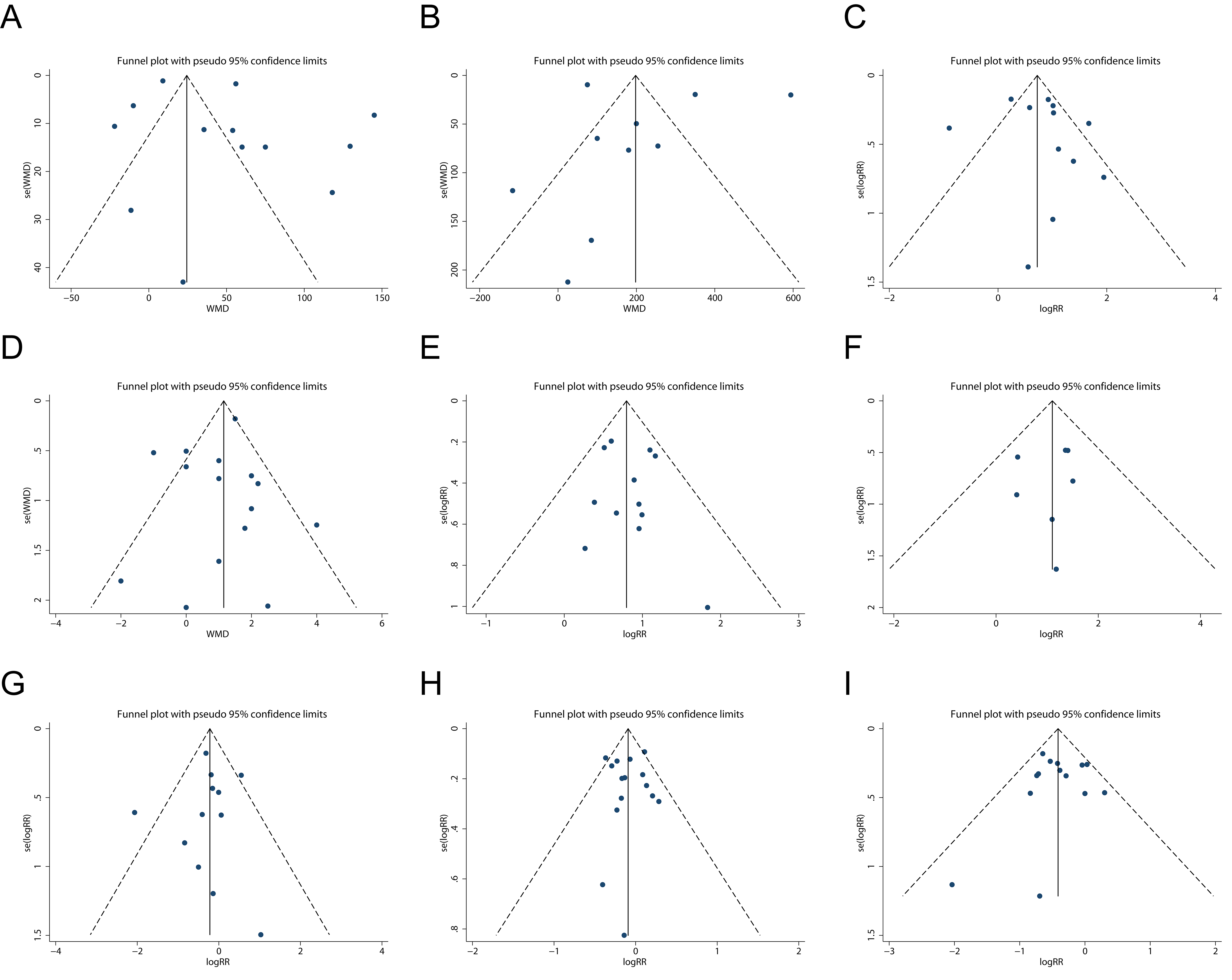


**Fig.S3** Funnel plots of short-term outcomes. Duration of operation (A), estimated blood loss (B), intraoperative blood transfusion (C), length of hospital stay (D), postoperative complications (E), 90‐day mortality (F), positive margin (G), intrahepatic recurrence (H), and repeat hepatectomy (I).
