# Supplementary material for: Clinical outcomes of parenchymal-sparing versus anatomic resection for colorectal liver metastases: a systematic review and meta-analysis
Source: World J Surg Oncol. 2023 Aug 8;21:241. doi: 10.1186/s12957-023-03127-1 (PMC10408219; doi:10.1186/s12957-023-03127-1)
Supplement: Supplementary file 2 — Additional file 2: Table S1. Quality of studies evaluated by modified Newcastle-Ottawa scale. [file 12957_2023_3127_MOESM2_ESM.docx]

| **Table S1** Quality of studies evaluated by modified Newcastle-Ottawa scale | | | | | | | | | |
| --- | --- | --- | --- | --- | --- | --- | --- | --- | --- |
| Study | Selection | | | | Comparability | Outcome | | | Quality score |
|  | Representativeness of cases | Representativeness of controls | Ascertainment of exposure | Definition of outcomes | Comparability of cases and controls | Assessment of outcome | Adequate follow-up time | Adequacy of follow-up |  |
| Andreou 2021 | ☆ | ☆ | ☆ | ☆ | ☆ | ☆ | ☆ | ☆ | 8 |
| Dam 2013 | ☆ | ☆ | ☆ | ☆ | ☆ | ☆ | ☆ | ☆ | 8 |
| DeMatteo 2000 | ☆ | ☆ | ☆ | ☆ | ☆ | ☆ | ☆ |  | 7 |
| Donadon 2017 | ☆ | ☆ | ☆ | ☆ | ☆ | ☆ | ☆ | ☆ | 8 |
| Finch 2007 | ☆ | ☆ | ☆ | ☆ | ☆ | ☆ | ☆ |  | 7 |
| Guzzetti 2008 | ☆ | ☆ | ☆ | ☆ | ☆ | ☆ | ☆ | ☆ | 8 |
| Hosokawa 2017 | ☆ | ☆ | ☆ | ☆ | ☆ | ☆ | ☆ | ☆ | 8 |
| Joechle 2020 | ☆ | ☆ | ☆ | ☆ | ☆ | ☆ | ☆ |  | 7 |
| Kokudo 2001 | ☆ | ☆ | ☆ | ☆ | ☆ | ☆ | ☆ |  | 7 |
| Lalmahomed 2011 | ☆ | ☆ | ☆ | ☆ | ☆ | ☆ | ☆ | ☆ | 8 |
| Lordan 2017 | ☆ | ☆ | ☆ | ☆ | ☆ | ☆ | ☆ | ☆ | 8 |
| Matsuki 2016 | ☆ | ☆ | ☆ | ☆ | ☆ | ☆ | ☆ | ☆ | 8 |
| Matsumura 2016 | ☆ | ☆ | ☆ | ☆ | ☆ | ☆ | ☆ |  | 7 |
| Memeo 2016 | ☆ | ☆ | ☆ | ☆ | ☆ | ☆ | ☆ | ☆ | 8 |
| Mise 2016 | ☆ | ☆ | ☆ | ☆ | ☆ | ☆ | ☆ | ☆ | 8 |
| Okumura 2019 | ☆ | ☆ | ☆ | ☆ | ☆ | ☆ | ☆ | ☆ | 8 |
| Pandanaboyana 2016 | ☆ | ☆ | ☆ | ☆ | ☆ | ☆ | ☆ | ☆ | 8 |
| Sarpel 2009 | ☆ | ☆ | ☆ | ☆ | ☆ | ☆ | ☆ | ☆ | 8 |
| She 2020 | ☆ | ☆ | ☆ | ☆ | ☆ | ☆ | ☆ | ☆ | 8 |
| Spelt 2018 | ☆ | ☆ | ☆ | ☆ | ☆ | ☆ | ☆ |  | 7 |
| Stewart 2004 | ☆ | ☆ | ☆ | ☆ | ☆ | ☆ | ☆ |  | 7 |
| Zorzi 2006 | ☆ | ☆ | ☆ | ☆ | ☆ | ☆ | ☆ |  | 7 |
