# Supplementary material for: Clinical outcomes of parenchymal-sparing versus anatomic resection for colorectal liver metastases: a systematic review and meta-analysis
Source: World J Surg Oncol. 2023 Aug 8;21:241. doi: 10.1186/s12957-023-03127-1 (PMC10408219; doi:10.1186/s12957-023-03127-1)
Supplement: Supplementary file 3 — Additional file 3. Supplemental file. The full electronic search strategy for each database. [file 12957_2023_3127_MOESM3_ESM.docx]

**Supplemental file. The full electronic search strategy for each database.**

**Pubmed** **database:**

("anatomical liver resection"[Title/Abstract] OR "anatomic resection"[Title/Abstract] OR "anatomic hepatectomy"[Title/Abstract] OR ("segmentectomy"[Title/Abstract] OR "subsegmentectomy"[Title/Abstract] OR "sectoriectomy"[Title/Abstract] OR "hemihepatectomy"[Title/Abstract] OR "trisectionectomy"[Title/Abstract] OR "semi-hepatectomy"[Title/Abstract] OR "lobectomy"[Title/Abstract] OR "liver lobectomy"[Title/Abstract] OR "liver lobe resection"[Title/Abstract] OR "Hepatic Lobectomy"[Title/Abstract] OR "Right hepatectomy"[Title/Abstract] OR "left lobectomy"[Title/Abstract] OR "left hepatectomy"[Title/Abstract] OR "right lobectomy"[Title/Abstract] OR "hepatolobectomy"[Title/Abstract] OR "Hepatic Lobectomy"[Title/Abstract] OR "anatomic liver resection"[Title/Abstract] OR "major hepatectomy"[Title/Abstract] OR "anatomical resection"[Title/Abstract] OR "major resection"[Title/Abstract] OR "anatomical hepatectomy"[Title/Abstract]) OR ("anatomic*"[Title/Abstract] OR "major"[Title/Abstract] OR "extended"[Title/Abstract] OR "exten*"[Title/Abstract] OR "central"[Title/Abstract])) AND (("parenchyma*"[All Fields] AND "sparing"[Title/Abstract]) OR "nonanatomic*"[Title/Abstract] OR "wedge"[Title/Abstract] OR "minor"[Title/Abstract] OR "segmental"[Title/Abstract] OR "limited"[Title/Abstract] OR "conservative"[Title/Abstract] OR ("parenchyma sparing resection"[Title/Abstract] OR "parenchyma sparing Hepatectomy"[Title/Abstract] OR "parenchymal preserving hepatectomy"[Title/Abstract] OR "parenchyma preserving resection"[Title/Abstract] OR ("wedge resection"[Title/Abstract] OR "minor hepatectomy"[Title/Abstract] OR "segmental resection"[Title/Abstract] OR "nonanatomic resection"[Title/Abstract] OR "limited resection"[Title/Abstract] OR "nodulectomy"[Title/Abstract] OR "minor liver resection"[Title/Abstract] OR "conservative resection"[Title/Abstract] OR "parenchymal sparing resection"[Title/Abstract] OR "parenchymal sparing Hepatectomy"[Title/Abstract] OR "parenchyma preserving hepatectomy"[Title/Abstract] OR "parenchymal preserving surgery"[Title/Abstract]))) AND ((("Colorectal Neoplasms"[MeSH Terms] OR "colorectal*"[MeSH Terms] OR "Colorectal Neoplasms"[Title/Abstract] OR "Colorectal cancer"[Title/Abstract] OR "Colorectal Carcinoma"[Title/Abstract] OR "colorectal*"[Title/Abstract]) AND ("Liver"[MeSH Terms] OR "Liver Neoplasms"[MeSH Terms] OR "Liver"[Title/Abstract] OR "hepatic"[Title/Abstract] OR "Liver Neoplasms"[Title/Abstract]) AND ("Neoplasm Metastasis"[MeSH Terms] OR "metastas*"[MeSH Terms] OR "neoplasm metastas*"[Title/Abstract] OR "metastas*"[Title/Abstract])) OR (((("Colorectal Neoplasms"[MeSH Terms] OR ("Colorectal"[All Fields] AND "neoplasms"[All Fields]) OR "Colorectal Neoplasms"[All Fields] OR ("Colorectal"[All Fields] AND "Cancer"[All Fields]) OR "Colorectal cancer"[All Fields]) AND ("Liver"[MeSH Terms] OR "Liver"[All Fields] OR "livers"[All Fields] OR "liver s"[All Fields])) AND "Neoplasm Metastasis"[MeSH Terms]) OR ((("Colorectal Neoplasms"[MeSH Terms] OR ("Colorectal"[All Fields] AND "neoplasms"[All Fields]) OR "Colorectal Neoplasms"[All Fields] OR ("Colorectal"[All Fields] AND "Cancer"[All Fields]) OR "Colorectal cancer"[All Fields]) AND ("Liver"[MeSH Terms] OR "Liver"[All Fields] OR "livers"[All Fields] OR "liver s"[All Fields])) AND "metastas*"[MeSH Terms]) OR (("Colorectal"[All Fields] AND ("Liver"[MeSH Terms] OR "Liver"[All Fields] OR "livers"[All Fields] OR "liver s"[All Fields])) AND "metastas*"[MeSH Terms]) OR "colorectal cancer liver metastas*"[Title/Abstract] OR "liver metastas*"[Title/Abstract] OR "hepatic metastas*"[Title/Abstract] OR "metastatic colorectal cancer"[Title/Abstract] OR "colorectal liver metastas*"[Title/Abstract] OR "CRLM"[Title/Abstract] OR "CLM"[Title/Abstract])) AND (("randomized controlled trial"[Publication Type] OR "controlled clinical trial"[Publication Type] OR "randomized"[Title/Abstract] OR "randomised"[Title/Abstract] OR "placebo"[Title/Abstract] OR "drug therapy"[MeSH Subheading] OR "randomly"[Title/Abstract] OR "trial"[Title/Abstract] OR "groups"[Title/Abstract] OR "comparative study"[Publication Type] OR "randomization"[Title/Abstract] OR "double blind procedure"[Text Word] OR "single blind procedure"[Text Word]) NOT ("animals"[MeSH Terms] NOT "humans"[MeSH Terms]))

**Cochrane Library database:**

ID Search Hits

#1 (hepatectomy):ti,ab,kw OR (Resection):ti,ab,kw OR (approach):ti,ab,kw OR ("liver resection"):ti,ab,kw OR (surgery):ti,ab,kw (Word variations have been searched) 320026

#2 MeSH descriptor: [Hepatectomy] explode all trees 658

#3 (methods):ti,ab,kw OR (therapy):ti,ab,kw OR ("liver metastases resection"):ti,ab,kw OR ("hepatic resection"):ti,ab,kw OR (treatment):ti,ab,kw (Word variations have been searched) 1347063

#4 (Anatomic*):ti,ab,kw OR (major):ti,ab,kw OR (exten*):ti,ab,kw OR (extended):ti,ab,kw OR (central):ti,ab,kw (Word variations have been searched) 478466

#5 #1 OR #2 OR #3 1403212

#6 #4 AND #5 439688

#7 (segmentectomy):ti,ab,kw OR (subsegmentectomy):ti,ab,kw OR (sectoriectomy):ti,ab,kw OR (hemihepatectomy):ti,ab,kw OR (trisectionectomy):ti,ab,kw (Word variations have been searched) 335

#8 (semi-hepatectomy):ti,ab,kw OR (lobectomy):ti,ab,kw OR ("liver lobectomy"):ti,ab,kw OR ("liver lobe resection"):ti,ab,kw OR ("Hepatic Lobectomy"):ti,ab,kw (Word variations have been searched) 1599

#9 ("Right hepatectomy"):ti,ab,kw OR ("left lobectomy"):ti,ab,kw OR ("left hepatectomy"):ti,ab,kw OR ("right lobectomy"):ti,ab,kw OR (hepatolobectomy):ti,ab,kw (Word variations have been searched) 74

#10 ("Hepatic Lobectomy"):ti,ab,kw OR ("anatomic liver resection"):ti,ab,kw OR ("major hepatectomy"):ti,ab,kw OR ("anatomical resection"):ti,ab,kw OR ("major resection"):ti,ab,kw (Word variations have been searched) 235

#11 ("anatomical hepatectomy"):ti,ab,kw OR (AR):ti,ab,kw OR (AH):ti,ab,kw OR (MH):ti,ab,kw (Word variations have been searched) 6849

#12 (MR):ti,ab,kw OR (ALR):ti,ab,kw OR (non-PSLR):ti,ab,kw 7181

#13 #7 OR #8 OR #9 OR #10 OR #11 OR #12 15948

#14 #6 OR #13 449373

#15 (nonanatomic*):ti,ab,kw OR ("parenchyma sparing"):ti,ab,kw OR (wedge):ti,ab,kw OR (minor):ti,ab,kw OR ("parenchymal sparing"):ti,ab,kw (Word variations have been searched) 27890

#16 (segmental):ti,ab,kw OR (limited):ti,ab,kw OR (conservative):ti,ab,kw OR ("parenchyma preserving"):ti,ab,kw OR ("parenchymal preserving"):ti,ab,kw (Word variations have been searched) 158238

#17 #15 OR #16 181980

#18 #5 AND #17 165230

#19 ("wedge resection"):ti,ab,kw OR ("minor hepatectomy"):ti,ab,kw OR ("segmental resection"):ti,ab,kw OR ("nonanatomic resection"):ti,ab,kw OR ("limited resection"):ti,ab,kw (Word variations have been searched) 430

#20 (nodulectomy):ti,ab,kw OR ("minor liver resection"):ti,ab,kw OR ("conservative resection"):ti,ab,kw OR ("parenchymal sparing resection"):ti,ab,kw OR ("parenchymal sparing Hepatectomy"):ti,ab,kw (Word variations have been searched) 30

#21 ("parenchyma preserving hepatectomy"):ti,ab,kw OR ("parenchymal preserving surgery"):ti,ab,kw OR (“nonanatomic hepatectomy”):ti,ab,kw OR (PSH):ti,ab,kw OR (PSR):ti,ab,kw (Word variations have been searched) 221

#22 (NAR):ti,ab,kw OR (PSLR):ti,ab,kw OR (NALR):ti,ab,kw OR (NAH):ti,ab,kw (Word variations have been searched) 326

#23 #19 OR #20 OR #21 OR #22 1002

#24 #18 OR #23 165707

#25 MeSH descriptor: [Colorectal Neoplasms] explode all trees 8895

#26 ("Colorectal Neoplasms"):ti,ab,kw OR (colorectal*):ti,ab,kw OR (rectal*):ti,ab,kw OR (colon*):ti,ab,kw OR ("Colorectal cancer"):ti,ab,kw (Word variations have been searched) 57718

#27 ("colorectal carcinoma"):ti,ab,kw OR (CRC):ti,ab,kw (Word variations have been searched) 5681

#28 #25 OR #26 OR #27 58324

#29 MeSH descriptor: [Liver] explode all trees 3382

#30 MeSH descriptor: [Liver Abscess] explode all trees 56

#31 MeSH descriptor: [Liver Neoplasms] explode all trees 3175

#32 (liver):ti,ab,kw OR (hepatic):ti,ab,kw OR ("Liver Neoplasms"):ti,ab,kw (Word variations have been searched) 75649

#33 #29 OR #30 OR #31 OR #32 75979

#34 MeSH descriptor: [Neoplasm Metastasis] explode all trees 5389

#35 ("Neoplasm Metastasis"):ti,ab,kw OR (metastas*):ti,ab,kw OR ("neoplasm metastas*"):ti,ab,kw OR (metastasis):ti,ab,kw OR (metastases):ti,ab,kw (Word variations have been searched) 26960

#36 #34 OR #35 27140

#37 #28 AND #33 AND #36 1908

#38 ("colorectal liver metastas*"):ti,ab,kw OR ("metastatic colorectal cancer"):ti,ab,kw OR ("hepatic metastas*"):ti,ab,kw OR ("liver metastas*"):ti,ab,kw OR ("colorectal cancer liver metastas*"):ti,ab,kw (Word variations have been searched) 3574

#39 ("colorectal cancer with liver metastas*"):ti,ab,kw OR ("colorectal cancer with liver metastas*"):ti,ab,kw OR ("colorectal cancer liver metastas*"):ti,ab,kw OR (CRLM):ti,ab,kw OR (CLM):ti,ab,kw (Word variations have been searched) 229

#40 #38 OR #39 3787

#41 #37 OR #40 5136

#42 #14 AND #24 AND #41 351

**Embase** **database:**

No. Query Results Results Date

#51. #9 AND #23 AND #47 AND #50 439

#50. #48 OR #49 3,649,051

#49. 'randomized controlled trial':ab,ti OR 'randomised':ab,ti OR 'randomized':ab,ti OR 'placebo':ab,ti OR 'rct':ab,ti OR 'controlled clinical trial':ab,ti OR 'randomly':ab,ti OR 'trial':ab,ti OR 'comparative study':ab,ti 2,071,472

#48. crossover AND ('procedure'/exp OR procedure) OR (double AND ('blind'/exp OR blind) AND ('procedure'/exp OR procedure)) OR (randomized AND controlled AND ('trial'/exp OR trial)) OR ('single blind' AND ('procedure'/exp OR procedure)) OR random* OR factorial* OR crossover* OR (cross AND over*) OR placebo* OR (doubl* AND adj AND blind*) OR (singl* AND adj AND blind*) OR assign* OR allocat* OR volunteer* OR rct 3,179,237

#47. #45 OR #46 77,232

#46. #28 AND #33 AND #40 33,738

#45. #41 OR #42 OR #43 OR #44 76,996

#44. colorectal AND ('liver'/exp OR liver) AND metastas* 32,950

#43. colorectal AND ('cancer'/exp OR cancer) AND ('liver'/exp OR liver) AND metastas* 32,292

#42. colorectal AND ('cancer'/exp OR cancer) AND ('liver'/exp OR liver) AND ('metastasis'/exp OR

metastasis) 31,571

#41. 'crlm':ab,ti OR 'clm':ab,ti OR 'colorectal liver metastas*':ab,ti OR 'metastatic colorectal cancer':ab,ti OR 'liver metastas* from colorectal carcinoma':ab,ti OR 'liver metastas* of colorectal cancer':ab,ti OR 'hepatic metastas*':ab,ti OR 'liver metastas*':ab,ti OR 'liver metastas* from colorectal cancer':ab,ti OR 'colorectal cancer liver metastas*':ab,ti OR 'liver metastas* in colorectal cancer':ab,ti OR 'colorectal cancer metastas* to the liver':ab,ti OR 'colorectal cancer with liver metastas*':ab,ti OR 'hepatic metastas* in colorectal carcinoma':ab,ti OR 'colorectal cancer hepatic metastas*':ab,ti 65,277

#40. #34 OR #35 OR #36 OR #37 OR #38 OR #39 882,954

#39. ('neoplasm'/exp OR neoplasm) AND metastas* 831,859

#38. 'neoplasm metastasis':ab,ti OR 'metastas*':ab,ti OR 'metastasis':ab,ti OR 'metastases':ab,ti OR 'neoplasm metastas*':ab,ti 571,167

#37. 'metastases'/exp OR metastases 774,456

#36. 'metastasis'/exp OR metastasis 831,697

#35. metastas* 854,587

#34. ('neoplasm'/exp OR neoplasm) AND ('metastasis'/exp OR metastasis) 813,962

#33. #29 OR #30 OR #31 OR #32 1,913,887

#32. 'liver':ab,ti OR 'hepatic':ab,ti OR 'liver neoplasms':ab,ti 1,356,405

#31. hepatic 483,758

#30. ('liver'/exp OR liver) AND ('neoplasms'/exp OR neoplasms) 520,051

#29. 'liver'/exp OR liver 1,845,300

#28. #24 OR #25 OR #26 OR #27 306,127

#27. 'colorectal neoplasms':ab,ti OR 'colorectal cancer':ab,ti OR 'colorectal carcinoma':ab,ti 182,519

#26. colorectal AND ('carcinoma'/exp OR carcinoma) 88,115

#25. colorectal AND ('cancer'/exp OR cancer) 296,287

#24. colorectal AND ('neoplasms'/exp OR neoplasms) 293,319

#23. #14 OR #22 1,821,327

#22. #15 OR #16 OR #17 OR #18 OR #19 OR #20 OR #21 15,839

#21. parenchyma* AND preserving AND resection 565

#20. parenchyma* AND preserving AND ('hepatectomy'/exp OR hepatectomy) 177

#19. parenchyma* AND sparing AND ('hepatectomy'/exp OR hepatectomy) 339

#18. parenchyma* AND sparing AND ('resection'/exp OR resection) 1,999

#17. nonanatomic* AND ('resection'/exp OR resection) 1,833

#16. nonanatomic* AND ('hepatectomy'/exp OR hepatectomy) 677

#15. 'wedge resection':ab,ti OR 'minor hepatectomy':ab,ti OR 'minor hepatectomy with parenchymal‐sparing approach':ab,ti OR 'non‐anatomical metastasectomy':ab,ti OR 'nonanatomical hepatectomy':ab,ti OR 'segmental resection':ab,ti OR 'hepatectomy of liver segments':ab,ti OR 'nonanatomic resection':ab,ti OR 'limited resection':ab,ti OR 'nodulectomy':ab,ti OR 'minor liver resection':ab,ti OR 'conservative resection':ab,ti OR 'parenchymal-sparing resection':ab,ti OR 'parenchymal-sparing hepatectomy':ab,ti OR 'parenchyma‐preserving hepatectomy':ab,ti OR 'parenchymal preserving surgery':ab,ti OR 'parenchyma sparing resection':ab,ti OR 'parenchyma sparing hepatectomy':ab,ti OR 'parenchymal preserving hepatectomy':ab,ti OR 'parenchyma preserving resection':ab,ti OR 'parenchymal preserving resection':ab,ti 12,063

#14. #10 OR #11 OR #12 OR #13 1,821,223

#13. 'nonanatomic*':ab,ti OR 'parenchyma-sparing':ab,ti OR 'wedge':ab,ti OR 'minor':ab,ti OR 'parenchymal‐sparing':ab,ti OR 'segmental':ab,ti OR 'limited':ab,ti OR 'conservative':ab,ti OR 'parenchyma‐preserving':ab,ti OR 'parenchymal-preserving':ab,ti 1,819,958

#12. parenchyma* AND preserving 1,232

#11. nonanatomic* 2,209

#10. parenchyma* AND sparing 2,443

#9. #1 OR #8 5,187,136

#8. #2 OR #3 OR #4 OR #5 OR #6 OR #7 185,903

#7. 'segmentectomy':ab,ti OR 'subsegmentectomy':ab,ti OR 'sectoriectomy':ab,ti OR 'hemihepatectomy':ab,ti OR 'trisectionectomy':ab,ti OR 'semi-hepatectomy':ab,ti OR 'lobectomy liver lobectomy':ab,ti OR 'liver lobe resection':ab,ti OR 'right hepatectomy':ab,ti OR 'left lobectomy':ab,ti OR 'left hepatectomy':ab,ti OR 'right lobectomy':ab,ti OR 'hepatolobectomy':ab,ti OR 'hepatic lobectomy':ab,ti OR 'anatomic liver resection':ab,ti OR 'hemihe-patectomy':ab,ti OR 'major hepatectomy':ab,ti OR 'anatomical resection':ab,ti OR 'major resection':ab,ti OR 'anatomical hepatectomy':ab,ti OR 'anatomical liver resection':ab,ti OR 'anatomic resection':ab,ti OR 'anatomic hepatectomy':ab,ti 17,289

#6. major AND ('hepatectomy'/exp OR hepatectomy) 21,996

#5. anatomical AND ('hepatectomy'/exp OR hepatectomy) 2,203

#4. anatomic AND ('hepatectomy'/exp OR hepatectomy) 1,343

#3. anatomical AND ('resection'/exp OR resection) 93,406

#2. anatomic AND ('resection'/exp OR resection) 70,937

#1. 'anatomic*':ab,ti OR 'exten*':ab,ti OR 'major':ab,ti OR 'extended':ab,ti OR 'central':ab,ti 5,151,929

**Web of science database：**

1 "TS=(Anatomic* OR exten* OR major OR extended OR central OR anatomical)"

2 "TS=(Resection OR hepatectomy OR approach OR liver resection OR surgery OR methods OR therapy OR hepatic metastases resection OR liver metastases resection OR hepatic resection)"

3 "#1 AND #2"

4 "TS=(hemihe-patectomy OR segmentectomy OR subsegmentectomy OR sectoriectomy OR hemihepatectomy OR trisectionectomy OR semi-hepatectomy OR lobectomy liver lobectomy OR liver lobe resection OR Hepatic Lobectomy OR Right hepatectomy OR left lobectomy OR left hepatectomy OR right lobectomy OR hepatolobectomy OR hepatic lobectomy OR anatomic liver resection OR hemihe-patectomy OR major hepatectomy OR anatomical resection OR major resection OR anatomical hepatectomy OR anatomical liver resection OR anatomic resection OR anatomic hepatectomy )"

5 "#4 OR #3"

6 "TS=(nonanatomic* OR parenchyma sparing OR wedge OR minor OR parenchymal sparing OR segmental OR limited OR conservative OR parenchyma preserving OR parenchymal preserving OR nonanatomic)"

7 "#6 AND #2"

8 "TS=(wedge resection OR minor hepatectomy OR minor hepatectomy with parenchymal‐sparing approach OR non‐anatomical metastasectomy OR nonanatomical hepatectomy OR segmental resection OR hepatectomy of liver segments OR nonanatomic resection OR limited resection OR nodulectomy OR minor liver resection OR conservative resection OR parenchymal-sparing resection OR parenchymal-sparing Hepatectomy OR parenchyma‐preserving hepatectomy OR parenchymal preserving surgery OR parenchyma sparing resection OR parenchyma sparing Hepatectomy OR parenchymal preserving hepatectomy OR parenchyma preserving resection OR parenchymal preserving resection OR nonanatomical resection )"

9 "#7 OR #8"

10 "TS=(CRLM OR CLM OR colorectal liver metastas* OR metastatic colorectal cancer OR liver metastas* from colorectal carcinoma OR liver metastas* of colorectal cancer OR hepatic metastas* OR liver metastas* OR liver metastas* from colorectal cancer OR colorectal cancer liver metastas* OR Liver Metastas* in Colorectal Cancer OR colorectal Cancer with Liver Metastas* OR colorectal cancer metastas* to the liver OR Colorectal Cancer With Liver Metastas* OR Hepatic Metastas* in Colorectal Carcinoma OR colorectal cancer hepatic metastas* OR Colorectal Cancer Liver Metastasis OR Colorectal Cancer Liver Metastas* OR colorectal liver metastas*)"

11 "#5 AND #9 AND #10"

12 "TS=(randomized controlled trial OR randomised OR randomized OR placebo OR RCT OR controlled clinical trial OR randomly OR trial OR comparative study)"

13 "#11 AND #12"
